# Comparison of Digital PCR and Quantitative PCR with Various SARS-CoV-2 Primer-Probe Sets

**DOI:** 10.4014/jmb.2009.09006

**Published:** 2020-12-25

**Authors:** Changwoo Park, Jina Lee, Zohaib ul Hassan, Keun Bon Ku, Seong-Jun Kim, Hong Gi Kim, Edmond Changkyun Park, Gun-Soo Park, Daeui Park, Seung-Hwa Baek, Dongju Park, Jihye Lee, Sangeun Jeon, Seungtaek Kim, Chang-Seop Lee, Hee Min Yoo, Seil Kim

**Affiliations:** 1 Microbiological Analysis Team, Biometrology Group, Korea Research Institute of Standards and Science (KRISS), Daejeon 34113, Republic of Korea; 2 National Research Laboratory of Molecular Microbiology and Toxicology, Department of Agricultural Biotechnology, Seoul National University, Seoul 08826, Republic of Korea; 3 Center for Convergent Research of Emerging Virus Infection, Korea Research Institute of Chemical Technology, Daejeon 34114, Republic of Korea; 4 College of Pharmacy, Chungnam National University, Daejeon 34134, Republic of Korea; 5 Department of Bio-Analytical Science, University of Science and Technology (UST), Daejeon 34113, Republic of Korea; 6 Research Center for Bioconvergence Analysis, Korea Basic Science Institute, Cheongju 28119, Republic of Korea; 7 Research Group of Food Processing, Korea Food Research Institute, Wanju-gun, Jeollabuk-do 55365, Republic of Korea; 8 Department of Predictive Toxicology, Korea Institute of Toxicology, Daejeon 34114, Republic of Korea; 9 Department of Biological Science, Chungnam National University College of Bioscience and Biotechnology, Daejeon 34134, Republic of Korea; 10 Zoonotic Virus Laboratory, Institut Pasteur Korea, Seongnam-si, Gyeonggi-do 13488, Republic of Korea; 11 Department of Internal Medicine, Jeonbuk National University Medical School, Jeonju 54986, Republic of Korea; 12 Biomedical Research Institute of Jeonbuk National University Hospital, Jeonju 54907, Republic of Korea

**Keywords:** COVID-19, SARS-CoV-2, reverse transcription-quantitative polymerase chain reaction (RT-qPCR), droplet digital PCR (ddPCR), nucleocapsid protein gene, envelope protein gene

## Abstract

The World Health Organization (WHO) has declared the coronavirus disease 2019 (COVID-19) as an international health emergency. Current diagnostic tests are based on the reverse transcription-quantitative polymerase chain reaction (RT-qPCR) method, which is the gold standard test that involves the amplification of viral RNA. However, the RT-qPCR assay has limitations in terms of sensitivity and quantification. In this study, we tested both qPCR and droplet digital PCR (ddPCR) to detect low amounts of viral RNA. The cycle threshold (C_T_) of the viral RNA by RT-PCR significantly varied according to the sequences of the primer and probe sets with in vitro transcript (IVT) RNA or viral RNA as templates, whereas the copy number of the viral RNA by ddPCR was effectively quantified with IVT RNA, cultured viral RNA, and RNA from clinical samples. Furthermore, the clinical samples were assayed via both methods, and the sensitivity of the ddPCR was determined to be equal to or more than that of the RT-qPCR. However, the ddPCR assay is more suitable for determining the copy number of reference materials. These findings suggest that the qPCR assay with the ddPCR defined reference materials could be used as a highly sensitive and compatible diagnostic method for viral RNA detection.

## Introduction

Coronaviruses have caused several notable respiratory disease outbreaks in humans, such as severe acute respiratory syndrome (SARS). The SARS coronavirus was the first fatal pathogenic human coronavirus to be identified and caused 8,096 infections and 774 deaths in 26 countries in 2003 [1]. Prior to the emergence of SARS-CoV, only two HCoVs (HCoV-229E and HCoV-OC43) were known, both of which caused mild respiratory symptoms [2]. Bats were the natural hosts of SARS-CoV, and civet cats were regarded as the intermediate host [3]. Extensive studies on bat coronaviruses showed that such coronaviruses could be potential human pathogens [4, 5]. MERS-CoV was first reported in 2012 and caused 2,494 infections and 858 deaths in 27 countries as of November 2019 (WHO, Middle East respiratory syndrome coronavirus (MERS-CoV)). MERS-CoV also originated from bat coronaviruses, and camels were the intermediate host [7].

According to the World Health Organization (WHO), the WHO China Country Office was informed of cases of pneumonia of unknown etiology in Wuhan City, Hubei Province, on December 31st, 2019 [8]. The novel coronavirus, currently termed SARS-CoV-2, was officially announced as the causative agent by the International Committee on Taxonomy of Viruses (ICTV) [9]. SARS-CoV-2, or severe acute respiratory syndrome coronavirus 2, has resulted in over fifty million laboratory-confirmed cases and over a million deaths as of November 2020 [10]. A viral genome sequence was released for immediate public health support via the community online resource virological.org on January 10th (Wuhan-Hu-1, GenBank accession number MN908947) [3], followed by four other genomes that were deposited on January 12th in the viral sequence database curated by the Global Initiative on Sharing All Influenza Data (GISAID). The genome sequences suggest the virus is closely related to the members of a viral species termed severe acute respiratory syndrome (SARS)-related CoV, a species defined by the agent of the 2002/03 outbreak of SARS in humans [4, 5]. The species also comprises of a large number of viruses mostly detected in Rhinolophid bats in Asia and Europe. For the past several decades, quantitative polymerase chain reactions (qPCR) have become the gold standard for quantifying relative gene expressions. On the other hand, the recently developed digital polymerase chain reaction (dPCR) enables a highly sensitive measurement and absolute quantitation of nucleic acids [11, 12]. Droplet dPCR (ddPCR) can provide absolute quantification of target DNA or RNA molecules without external reference materials. The PCR reaction mixture with target nucleic acid molecules are portioned into thousands of nanoliter-sized water-in-oil emulsion droplets. These droplets are amplified with PCR, and each individual droplet is analyzed. The droplets with a template are counted as PCR-positive droplets, and the droplets without template are PCR-negative droplets. The absolute quantification of target nucleic acids can be done based on the number of positive and negative droplets. ddPCR has an advantage for the clinical diagnosis of SARS-CoV-2 to reduce the false negative results [13], which could be highly sensitive compared to real time PCR (RT-PCR) [14-17]. Moreover, there are some research groups that have analyzed many samples from COVID-19 confirmed patients by droplet digital PCR (ddPCR) and RT-PCR based on two target genes (ORF1ab and N). The overall concept looks similar to analyzing a large number of COVID-19 patient samples using digital PCR [18, 19]. However, none of these groups applied the ddPCR reference value using the reference material in the patient sample to measure an accurate quantification and to achieve diagnostic reliability [13,20-24]. DNA or RNA quantities should be metrologically traceable to a reference [25, 26]. As of this writing, multiple nucleic acid quantitation methods have been developed, such as chemical analysis methods based on isotope-dilution mass spectrometry (IDMS), capillary electrophoresis (CE), and enumeration-based flow cytometric method (FCM) counting [27, 28]. These methods can be accurately calibrated with solutions of nucleic acids. In addition, an international comparison study was performed between national metrology institutes (NMI) using the ddPCR method [29]. Recently, the droplet digital PCR (ddPCR) method has emerged as a powerful analytical technique for clinical utility [30, 31]. For example, ddPCR can be used for the rapid enumeration of viral genomes and particles [32-34].

In the present case of SARS-CoV-2, virus isolates or samples from infected patients have yet to be made available to the international public health community. In this study, we concluded that the optimized conditions are required to increase the precision of ddPCR to develop reference materials with matrix conditions.

## Materials and Methods

### Construction of RNA Standards

Full-length N and E genes of SARS-CoV-2 (GeneBank: MN908947) were synthesized and cloned in the pET21a plasmid. The plasmids were amplified with the T7 promoter primer (5’-TAATACGACTCACTATAGGG-3’) and T7 terminator primer (5’-GCTAGTTATTGCTCAGCGG-3’). The amplicons were spectrophotometrically quantified at 260 nm. A total of 200 ng of the PCR product was used for the in vitro transcription (MEGAscript T7 Transcription kit; Thermo Fisher Scientific, USA), which was performed at 37°C overnight in a 20 μl reaction mixture containing 2 μl of reaction buffer, 2 μl of each nucleoside triphosphate, and 2 μl of enzyme mix. The template DNAs were removed by digestion with 2 U of Turbo DNase I for 15 min at 37°C. The RNAs were precipitated by adding 6 μl of 3 M sodium acetate and 150 μl of 98% ethanol, which was followed by a subsequent incubation at -20°C for 30 min. After 15 min of centrifugation at 17,000 rpm, the supernatant was removed and 200 μl of 70% ethanol was added. After another 10-min centrifugation at 17,000 rpm, the supernatant was removed, and the pellet was dissolved in 20 μl of RNase-free H_2_O (Takara Bio Inc., Japan). Quantitation of the RNAs was performed spectrophotometrically at 260 nm. The measurement of the RNA concentrations was performed in duplicate, and the concentration was then converted to the molecule number [35].

### Cell and Virus RNA Extraction

The human cell lines were obtained from the relevant culture collections Vero E6 cells (ATCC CRL-1586) and MRC-5 cells (ATCC CCL-171) were obtained from the American Type Culture Collection (ATCC), and Huh-7 cells (JCRB 0403) were obtained from the Japan Cell Research Bank (JCRB). The cells were cultured at 37°C in a 5% CO_2_ incubator unless described otherwise.

The clinical isolate SARS-CoV-2 (NCCP 43326) was obtained from the National Culture Collection for Pathogens (NCCP). The strains were introduced into the Vero E6 cells (ATCC CRL-1586) and maintained in Dulbecco’s modified Eagle medium (DMEM) supplemented with 2% (v/v) fetal bovine serum (FBS) and 1%antibiotic-antimycotic solution (Thermo Fisher Scientific). The SARS coronavirus HKU-39849 (GenBank:AY278491.2, provided by Dr. Malik Peiris of the University of Hong Kong) was inoculated into the Vero E6 cells. Cytopathic effects were observed two days after inoculation and the viral titer was determined via a plaque assay. The patient-derived isolate MERS-CoV strain KNIH/002_05_2015 [36] was obtained from the KNIH and inoculated into cultured Huh-7 cells. The Huh-7 cells were maintained in Dulbecco’s modified Eagle’s medium (DMEM, HyClone, USA) supplemented with 10% fetal bovine serum (FBS, HyClone, USA) at 37°C in a 5%CO_2_ incubator. HCoV OC43 (ATCC VR1588) was obtained from the ATCC and inoculated into the MRC-5 cells. The culture media of the MRC-5 cells were Gibco Minimum Essential Media (MEM; Thermo Fisher Scientific) supplemented with 10% heat-inactivated Fetal Bovine Serum (FBS), 1% Sodium Pyruvate, 1% penicillin, and 1%non-essential amino acids. The inoculated cells were cultured with virus growth medium (MEM with 2% heat-inactivated FBS, 1% penicillin, and 1% non-essential amino acids) at 33°C. The supernatant of the culture medium was stored at -80°C until RNA extraction. The viral RNAs of SARS-CoV-2, MERS-CoV and HCoV-OC43 were extracted using the QIAamp Viral RNA Mini Kit (Qiagen, Germany) according to the manufacturer’s instructions. The viral RNA of SARS-CoV was extracted from the culture medium using the MagMAX-96 viral RNA isolation kit (Thermo Fisher Scientific) according to the manufacturer's instructions.

### Clinical Samples and RNA Preparation

The clinical samples used in this study were collected from subjects according to registered protocols approved by the Institutional Review Board (IRB) of Jeonbuk National University Hospital with all patients having signed written informed consent forms (IRB registration number: CUH 2020-02-050-008). The clinical characteristics of the patients are shown in Table S1. Upper respiratory tract specimens (naso- and oropharyngeal swabs) from COVID-19 patients were suspended in a transport medium (eNAT; Copan, USA) and stored at -80°C until use. The RNA extraction from the clinical samples was performed using the QIAamp Viral RNA Mini Kit (Qiagen) according to the manufacturer’s instructions. The extracted viral RNA was used as a template for qPCR assays and cDNA synthesis. The qPCR assays with undiluted RNA were done using the Allplex SARS-CoV-2 Assay kit (Seegene, Korea) according to the manufacturer’s instruction. The Ct values from extracted RNA were shown in Table S1.

### Primer and Probes

The primer-probe sets used in this study are listed in Table 1. For the detection of SARS-CoV-2, primer-probe sets of from the Centers for Disease Control and Prevention (CDC, USA), the University of Hong Kong (HKU, Hong Kong), the National Institute of Infectious Disease Department of Virology III (NIID, Japan), and the National Institute of Health (NIH, Thailand) were selected according to laboratory guidance from WHO [37]. The specific primer-probe sets for MERS-CoV and HCov-OC43 were used for the quantification of the viral RNA genomes [38, 39]. All primers and probes were synthesized by Neoprobe (Korea). All probes were labeled with the reporter molecule 6-carboxyfluorescein (FAM) at the 5'-end and with the quencher Black Hole Quencher 1 (BHQ-1) at the 3'-end.

### cDNA Synthesis of Viral RNA Genomes

The cDNAs of the clinical and cultured (SARS-CoV-2, SARS-CoV, MERS-CoV, and HCoV-OC43) viral RNA genomes were synthesized using the LunaScript RT SuperMix Kit (New England Biolabs, USA). The reaction mixture had a volume of 20μl and consisted of 4μl of 5X LunaScript RT Supermix, 1μl of RNA template, and 15 μl of distilled water. The reactions were carried out according to the manufacturer’s instructions. The synthesized cDNAs were serially diluted with distilled water and used as templates for qPCR and ddPCR without purification.

### qPCR and Droplet Digital PCR Measurement

The templates were serially diluted to a few copy numbers/μl. To remove the bias from different reverse transcription steps, the same cDNAs from the viral RNA were used as templates for both qPCR and ddPCR. RT-qPCR analysis was performed on the StepOne and StepOnePlus Real-Time PCR system (Thermo Fisher Scientific). The reaction mixture had a volume of 25μl and consisted of 12.5μl of maxima Probe/ROX qPCR master mix (2X) (Thermo Fisher Scientific), 1μl of cDNA template, 1μl of 10μM forward primer, 1μl of 10μM reverse primer, 1μl of 5 μM probe labeled with FAM, and 8.5 μl of distilled water. The ddPCR analysis was performed using a QX200 system (BioRad Laboratories, USA). The reaction mixture had a volume of 20μl and consisted of 10μl of ddPCR supermix for probes (BioRad Laboratories), 1μl of cDNA template, 1 μl of 10μM forward primer, 1μl of 10μM reverse primer, 1μl of 5 μM probe labeled with FAM, and 6 μl of distilled water. All valid copy numbers were selected according to the manufacturer's instructions [40].

### Data Analysis

The qPCR data were initially analyzed using the StepOne software (Thermo Fisher Scientific). Raw data (*i.e.*, the fluorescence values for Ct) were exported from the StepOne software to Microsoft Excel 2016. A series of diluted templates were used to determine the Ct value, which can establish a standard curve for evaluating the reaction efficiency. Droplet fluorescence data were initially analyzed using the QuantaSoft software (BioRad Laboratories). Raw data (*i.e.*, the fluorescence values for the droplets) were exported from the QuantaSoft software to Microsoft Excel 2016. Depending on the separation of positive and negative droplets, an objective separation value k was automatically calculated.

## Results

### Quantification of Viral and IVT RNAs

**Fig. 1:**
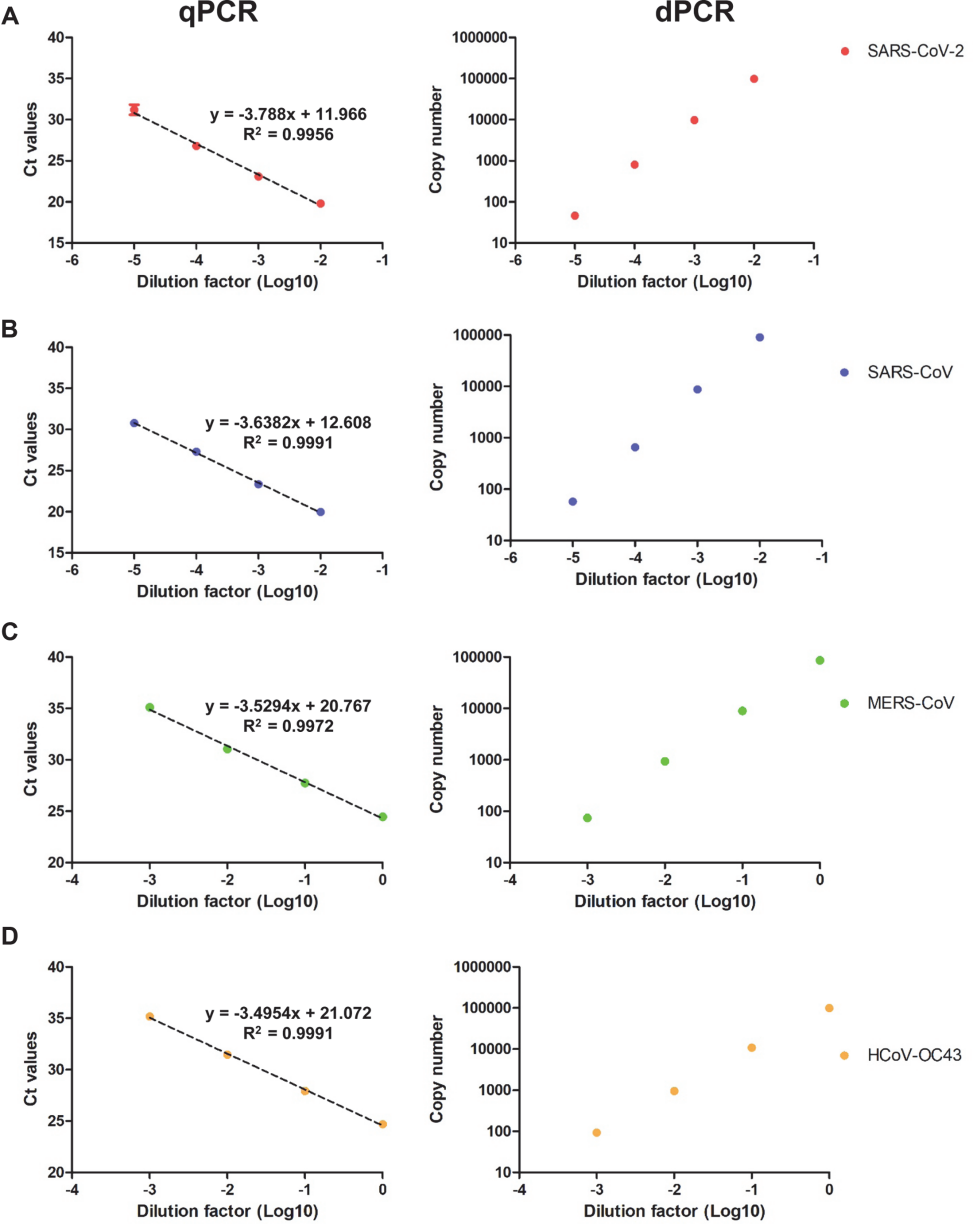
Detection of four viruses based on E gene assays by qPCR and ddPCR. (**A**) and (**B**): The Ct value and copy number of SARS-CoV-2 and SARS-CoV using the E sarbecovirus assay. (**C**) and (**D**): The Ct value and copy number of MERS and OC43 virus using the upE and OC43_E assay, respectively. All template were performed in triplicate and points indicates average value.

**Fig. 2:**
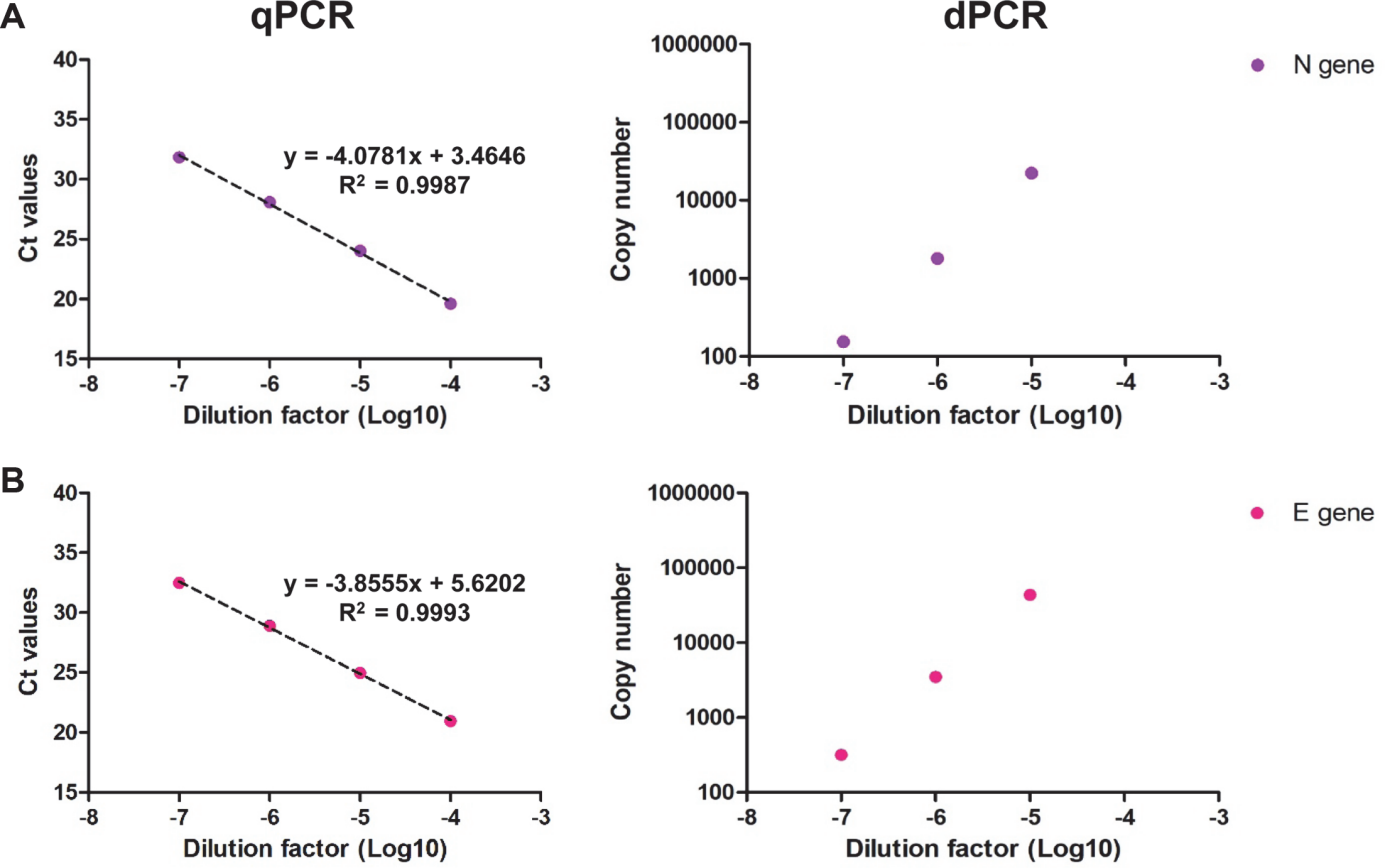
Detection of the IVT gene by qPCR and ddPCR. (**A**): The Ct value and copy number of the IVT N gene using the 2019-nCoV_N2 assay. (**B**): The Ct value and copy number of the IVT E gene using the E sarbecovirus assay. All template were performed in triplicate and points indicates average value.

In this study, the primer-probe sets of each E gene were used to quantify the viral genomic RNA of SARS-CoV-2 and related coronaviruses in triplicate (Fig. 1 and Table 1) [37-39,41]. SARS-CoV, MERS-CoV, and HCoV-OC43 were used a negative control for the assessment of SARS-CoV-2 specific primer-probe sets. The copy numbers of SARS-CoV-2, SARS-CoV, MERS-CoV, and HCoV-OC43 viral RNA were measured using ddPCR, and the measured values of the viral genomic RNA were 8.04 × 10^5^, 6.52 × 10^5^, 9.37 × 10^4^, and 9.57 × 10^4^ copies/μl, respectively. The standard curves of qPCR and ddPCR are linear, indicating the amplification efficiency of both methods is consistent (Fig. 1). The quantified viral genomic RNAs of SARS-CoV, MERS-CoV, and HCoV-OC43 were used as negative templates for SARS-CoV-2 specific assays. To assess the quantification of the viral genomic RNA and partial synthetic viral RNA, in vitro transcript (IVT) N and E gene RNA were measured via qPCR and ddPCR in triplicate (Fig. 2). The copy numbers of N IVT RNA and E IVT RNA were measured as 1.8 × 10^9^ and 3.47 × 10^9^, respectively. The amplification efficiencies were calculated from the standard curves of SARS-CoV-2 viral RNA and IVT RNAs. In the qPCR reactions, each E gene primer-probe sets targeting the SARS-CoV-2, SARS-CoV, MERS-CoV, and HCoV-OC43 showed Ct values of 28.64, 27.96, 26.96, and 27.69, respectively. In addition, the ddPCR reactions with each E gene primer-probe set targeting SARS-CoV-2, SARS-CoV, MERS-CoV, and HCoV-OC43 showed copy numbers of 2.68 × 10^6^, 4.69 × 10^6^, 7.85 × 10^5^, and 2.66 × 10^5^, respectively.

### Primer-Probes Validation

**Fig. 3:**
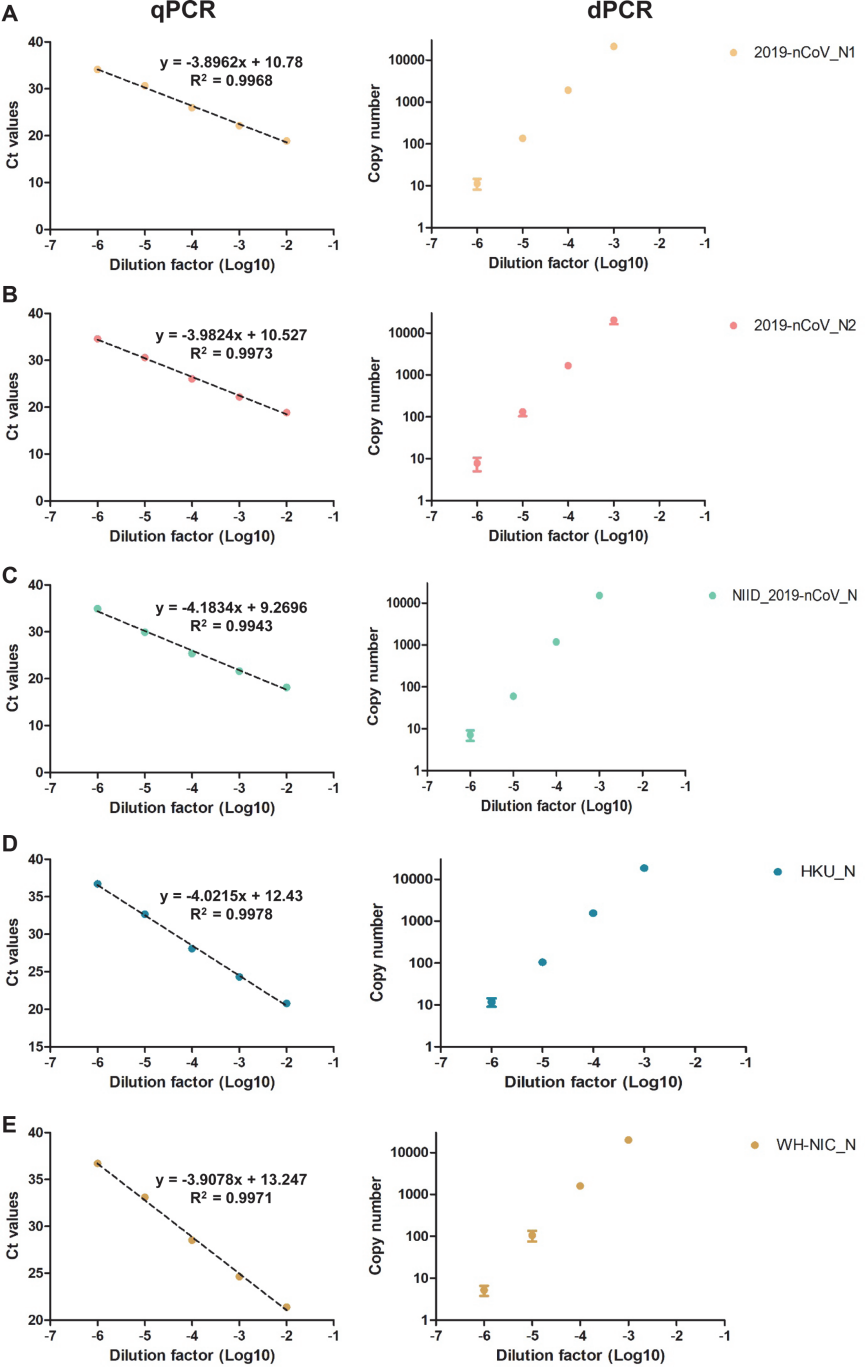
Comparison of the N gene primer-probe sets for the qPCR and ddPCR methods. For the comparison of viral SARS-CoV-2, primer-probe sets from the Centers for Disease Control and Prevention (CDC, USA) (**A** and **B**), the National Institute of Infectious Disease Department of Virology III (NIID, Japan) (**C**), the University of Hong Kong (HKU, Hong Kong) (**D**), and the National Institute of Health (NIH, Thailand) (**E**) were used based on the results of Table 4. All template were performed in triplicate and points indicates average value.

To assess the specificity and sensitivity of the five SARS-CoV-2 specific N gene targeting primer-probe sets (2019-nCoV_N1, 2019-nCoV_N2, NIID_2019-nCoV_N, HKU-N, WH-NIC N, the assays were done with SARS-CoV-2 RNA and other human pathogenic corona viral RNAs (SARS-CoV, MERS-CoV, and HCoV-OC43). The qPCR and ddPCR reactions using the N gene targeting primer-probes sets were positive in the SARS-CoV-2 genomic RNA in triplicate (Fig. 3 and Table 3). The reactions were negative with the other betacorona viral genomic RNAs such as SARS-CoV, MERS-CoV, and HCoV-OC43. The reaction with the E primer-probe sets was positive for each target viral RNA. The reactions using *sarbecovirus* (Betacoronavirus lineage B) targeting the HKU-N and E-sarbeco primer-probe sets were positive with both SARS-CoV and SARS-CoV-2 genomic RNA. The results showed that both qPCR and ddPCR reactions using the N primer-probe sets are specific to the SARS-CoV-2, and the *sarbecovirus* targeting primer-probe sets are also specific to SARS-CoV and SARS-CoV-2.

### IVT RNAs as a Standard for RT-qPCR

The R^2^ values of the viral RNA and IVT RNA (N genes) standard curve from the RT-qPCR assays were calculated as 0.9973 and 0.9987, respectively, whereas the R^2^ values of the viral RNA and IVT RNA (E genes) standard curve from the RT-qPCR assays were calculated as 0.9956 and 0.9993, respectively (Figs. 1 and 2 and Tables 2 and 3). These values indicate that the standard curves of both the viral and IVT RNAs are linear and accurate. The amplification efficiency of both the viral and IVT RNAs were calculated from the slopes of the standard curves. The efficiencies of both the viral and IVT RNAs according to the target genes were similar (Figs. 1 and 2). The similar amplification efficiencies show that the quantified IVT RNA can be used as a standard for RT-qPCR measurement of viral RNA.

### Comparison of qPCR and ddPCR Assays for COVID-19 Clinical Samples

Both the qPCR and ddPCR assays were tested with five SARS-CoV-2 positive and three SARS-CoV-2 negative clinical samples. The clinical characteristics of the confirmed COVID-19 patients with symptoms are summarized in Table S1. The positive patients were determined in clinics with the qPCR assay. The results from the positive samples showed that both qPCR and ddPCR can detect up to approximately 10 copy/μl of SARS-CoV-2 genomic RNA (Table 5). The control assays without templates did not produce any signals from both the qPCR and ddPCR assays. However, the qPCR and ddPCR assays with three negative patient samples (N1, N3, and N5) showed a high Ct value or small amount of copy number in some reactions (Table 4). According to the qPCR and ddPCR results with templates of very low concentrations, the signals from assays with a template of very low concentration can be difficult to distinguish from background signals. These results indicated that the sensitivity of ddPCR can be equal to or more than that of the optimized qPCR.

## Discussion

The outbreak of COVID-19 is a pandemic threat caused by the emergence of SARS-CoV-2, a newly discovered human coronavirus [42, 43]. SARS-CoV-2 has four structural proteins, known as the S (spike), E (envelope), M (membrane), and N (nucleocapsid) proteins [44]. The SARS-CoV-2 S protein binds with angiotensin-converting enzyme 2 (ACE2) with high-affinity, which leads to clinical features of pneumonia in many patients [45, 46]. SARS-CoV-2 is highly contagious and has quickly spread across the world, which further emphasizes the essential role of diagnostics in the control of communicable diseases [47].

Currently, a reliable test (also the most widely used) for detecting acute infections of SARS-CoV-2 is the reverse transcription-quantitative polymerase chain reaction (RT-qPCR) method [48]. Multiple national laboratories have developed assays targeting conserved regions that could potentially detect pancoronaviruses [49, 50]. A previous study provided information such as the specific primer and probe sequence, thermal profile, and reagents for the RT-qPCR to optimize the reaction conditions for the detection of SARS-CoV-2 RNA [51]. ddPCR is a method that improves upon conventional PCR methods that can individually amplify and directly quantify pathogen RNA or DNA [52]. Due to the principle of ddPCR, the signal from each droplet with a template can be clearly distinguished from the noise from the droplet without a template. Several studies have compared RT-qPCR and ddPCR for the quantification of viral genomes [53-55].

In this study, SARS-CoV-2 RNA concentrations were measured using the qPCR and ddPCR methods. The qPCR results showed that the Ct value of a given template was greatly dependent on the individual primer-probe sets. However, the copy numbers determined by ddPCR were relatively stable and reliable in the quantification of viral RNA samples, regardless of the primer-probe sets. A previous study reported that qPCR and ddPCR results were consistent for 95 positive samples and that there was a high correlation between the Ct values of qPCR and the copy number values of ddPCR among patient samples [56]. Although the Ct values of each primer-probe set exhibited variance, the copy numbers from ddPCR showed that N genes are more abundant than E genes. These findings are also consistent with another previous study, indicating N genes are good targets for SARS-CoV-2 diagnostics [57].

The qPCR method requires the use of a standard curve to obtain quantitative measurements of an unknown sample [58]. However, in the case of ddPCR, the distribution of a target-specific amplification into partitions is calculated using the Poisson distribution, enabling the absolute quantification of the target gene based on the ratio of positive against all partitions at the end of the reaction without dependence on an external standard [59]. In addition to these advantages, our results revealed that ddPCR is a sensitive and accurate method for SARS-CoV-2 detection in low viral copies (Fig. 3). Further experiments should be performed even if patients are cured of COVID-19, and such tests should be conducted using ddPCR to lower the limit of detection (LOD) to be able to detect low amounts of viral RNA.

The Coronavirus Standards Working Group led by the Joint Initiative for Metrology in Biology (JIMB) is conducting international collaborative experiments to develop guidelines for the purpose of evaluating and establishing the availability of common and appropriate standards, diverse reference materials, validation tests, and reference measurement protocols that can enable reliable COVID-19 testing [60, 61]. Our results serve as a guide for the standardization of analytical methods and the development of reference materials that are required for accurate COVID-19 diagnosis based on both RT-qPCR and ddPCR. Although, ddPCR-based diagnostics can serve as a companion method to the current standard RT-qPCR to provide sensitive and accurate quantitative results for emergency testing that can be adopted for clinical use, there are some drawbacks. The narrow dynamic range and high capital and operational cost of ddPCR are the reasons preventing the deployment of ddPCR in clinical fields. Due to the nature of ddPCR, the precise quantification can be done with a decent number of positive droplets. ddPCR can be a precise diagnostics method, but there are some drawback. Therefore, the combination of both qPCR and ddPCR could be better methods for the SARS-CoV-2 diagnostics; qPCR assay with ddPCR defined reference materials could be precise and practical assays for pathogen detection.

## Conclusion

Our study demonstrated that the Ct values of samples from qPCR assays were significantly variable depending on the sequences of primer and probe sets, whereas the copy numbers of the samples from the ddPCR assays were hardly affected by the sequences of the primer and probe sets. These results indicate that ddPCR can be performed with sub-optimal primer-probe sets without the loss of sensitivity. A comparison of the standard curves of viral RNA and IVT RNA showed that the IVT RNA quantified by ddPCR could be used as a standard for the absolute quantification of the qPCR assays. The results from the clinical samples showed that the ddPCR method could be used as a sensitive and quantitative diagnostic assay. Although the qPCR produced different Ct values using different primer-probes sets, the ddPCR results obtained using different primer-probe sets were compatible with each other. However, the narrow dynamic range and high cost of ddPCR are drawbacks for its use a clinical diagnostic method. Therefore, the deployment of qPCR assays with ddPCR-based reference materials could produce highly sensitive and quantitative results that are compatible between different laboratories.

## Supplemental Materials



Supplementary data for this paper are available on-line only at http://jmb.or.kr.

## Figures and Tables

**Table 1:** Information of primers and probes used in the study.

Institute	Gene	Name	Type^*^	Sequence (5’ → 3’)	Taxon	Ref.
CDC (USA)	N	2019-nCoV_N1	F	GACCCCAAAATCAGCGAAAT	SARS-CoV-2	[37]
			R^**^	TCTGGTTACTGCCAGTTGAATCTG		
			P^+^	ACCCCGCATTACGTTTGGTGGACC		
		2019-nCoV_N2	F	TTACAAACATTGGCCGCAAA	SARS-CoV-2	
			R	GCGCGACATTCCGAAGAA		
			P	ACAATTTGCCCCCAGCGCTTCAG		
NIID (Japan)		NIID_2019-nCoV_N	F	AAATTTTGGGGACCAGGAAC	SARS-CoV-2	
			R	TGGCAGCTGTGTAGGTCAAC		
			P	ATGTCGCGCATTGGCATGGA		
NIH (Thailand)		WH-NIC N-R	F	CGTTTGGTGGACCCTCAGAT	SARS-CoV-2	
			R	CCCCACTGCGTTCTCCATT		
			P	CAACTGGCAGTAACCA		
HKU (Hong Kong)		HKU-N	F	TAATCAGACAAGGAACTGATTA	Sarbeco	
			R	CGAAGGTGTGACTTCCATG		
			P	GCAAATTGTGCAATTTGCGG		
Charité (Germany)	E	E_Sarbeco	F	ACAGGTACGTTAATAGTTAATAGCGT	Sarbeco	[41]
			R	ATATTGCAGCAGTACGCACACA		
			P	ACACTAGCCATCCTTACTGCGCTTCG		
University of Bonn Medical Centre (Germany)		upE	F	GCAACGCGCGATTCAGTT	MERS-CoV	[38]
			R	GCCTCTACACGGGACCCATA		
			P	CTCTTCACATAATCGCCCCGAGCTCG		
University of Leuven (Belgium)		OC43	F	ATGTTAGGCCGATAATTGAGGACTAT	HCoV-OC43	[39]
			R	AATGTAAAGATGGCCGCGTATT		
			P	CATACTCTGACGGTCACAAT	

*F: forward primer, R: reverse primer, P: Probe

**Table 2:** Validation of the SARS-CoV-2 specific N gene targeting primer-probe sets used in the study.

Template	SARS-CoV-2 (15 pg/μl)		SARS-CoV (8.7 pg/μl)		MERS-CoV (72 pg/μl)		HCoV-OC43 (60 pg/μl)	

Primer-probes	qPCR (Ct)	ddPCR (copy/μl)	qPCR (Ct)	ddPCR (copy/μl)	qPCR (Ct)	ddPCR (copy/μl)	qPCR (Ct)	ddPCR (copy/μl)
2019-nCoV_N1	26.30	6123	Undetermined	0	Undetermined	0	Undetermined	0
2019-nCoV_N2	26.57	5340	Undetermined	2.2	Undetermined	4.3	39.09	4.8
NIID_2019-nCoV_N	26.89	3993	Undetermined	0	Undetermined	0	Undetermined	0
HKU-N	27.57	4863	27.24	6030	Undetermined	0	Undetermined	0.8
WH-NIC N	28.91	6153	Undetermined	0	Undetermined	0	Undetermined	0.5

*All template were performed in triplicate and data in table indicates average value.

**Table 3:** Comparison of the N-gene targeting SARS-CoV-2 qPCR and ddPCR assays.

Dilution factor	2019-nCoV_N1		2019-nCoV_N2		NIID_2019-nCoV_N		WH-NIC N		HKU-N	

	qPCR	ddPCR	qPCR	ddPCR	qPCR	ddPCR	qPCR	ddPCR	qPCR	ddPCR
10^-2^	18.89	S^*^	18.87	S	18.14	S	21.40	S	20.79	S
10^-3^	22.16	21173	22.19	20340	21.63	15173	24.65	19993	24.31	18473
10^-4^	25.99	1907	26.06	1659	25.40	1177	28.52	1599	28.09	1553
10^-5^	30.66	135	30.60	132	29.93	60	33.11	105	32.68	105
10^-6^	34.12	11	34.57	8	34.91	7	36.71	5	36.71	12
R^2^	0.9968		0.9973		0.9943		0.9971		0.9978	

*Saturated

**All template were performed in triplicate and data in table indicates average value.

**Table 4:** Comparison of the N-gene targeting SARS-CoV-2 qPCR and ddPCR assays on negative patient sample.

Primer-Probes	2019-nCoV_N2 primer-probe set		WH-NIC N primer-probe set	

Template	qPCR (Ct)	ddPCR (copy/μl)	qPCR (Ct)	ddPCR (copy/μl)
N1 (153ng/μl)	Undetermined	2.8	Undetermined	0
N3 (101ng/μl)	Undetermined	4.3	Undetermined	0
N5 (243ng/μl)	Undetermined	0.5	Undetermined	0.5

*All template were performed in triplicate and data in table indicates average value.

**Table 5:** Results of the Ct values by qPCR and copy number by ddPCR on patient sample.

Primer-probe	2019-nCoV_N2 primer-probe set									

Template	P1		P2		P3		P4		P5	

Dilution factor	qPCR (Ct)	ddPCR (copy/μl)	qPCR (Ct)	ddPCR (copy/μl)	qPCR (Ct)	ddPCR (copy/μl)	qPCR (Ct)	ddPCR (copy/μl)	qPCR (Ct)	ddPCR (copy/μl)

10^-1^	26.87	1051	35.03	7.2	35.15	2.4	Undetermined	0	35.05	7.53
10^-2^	30.52	82	37.03	1.33	Undetermined	0.73	Undetermined	0.53	Undetermined	1.2
10^-3^	33.81	5.93	Undetermined	0.67	Undetermined	0.6	Undetermined	0	Undetermined	1.07

Primer-probe	WH-NIC N primer-probe set									

Template	P1		P2		P3		P4		P5	

Dilution factor	qPCR (Ct)	ddPCR (copy/μl)	qPCR (Ct)	ddPCR (copy/μl)	qPCR (Ct)	ddPCR (copy/μl)	qPCR (Ct)	ddPCR (copy/μl)	qPCR (Ct)	ddPCR (copy/μl)

10^-1^	28.77	836.00	36.11	5	35.88	6.80	Undetermined	1.40	35.86	7.67
10^-2^	32.25	107.33	38.54	3	38.52	1.93	Undetermined	0.00	37.02	1.87
10^-3^	35.95	10.40	Undetermined	2.40	38.27	2.07	38.44	1.27	Undetermined	0.47

*The initial concentration of RNA templates were 46.9, 21.1, 27.5, 17.2, and 15.9 ng/μl, respectively.

**All template were performed in triplicate and data in table indicates average value.

^+^Only one of the triplicate was positive (single droplet)
