# Supplementary material for: Comparison of Digital PCR and Quantitative PCR with Various SARS-CoV-2 Primer-Probe Sets
Source: J Microbiol Biotechnol. 2020 Dec 25;31(3):358–67. doi: 10.4014/jmb.2009.09006 (PMC9705847; doi:10.4014/jmb.2009.09006)
Supplement: Supplementary file 1 [file jmb-31-3-358-supple.pdf]

J. Microbiol. Biotechnol.
https://doi.org/10.4014/jmb.2009.09006

Supplementary Table 1. Clinical characteristics of laboratory-confirmed patients with COVID-19

	Patient 1	Patient 2	Patient 3	Patient 4	Patient 5
Age (years)/Sex	72/M	57/M	72/F	48/M	51/F
Fever	-	+	+	+	+
Chills	-	+	-	-	-
Cough	+	+	+	-	+
Dyspnea	-	+	+	-	+
Diarrhea	-	-	-	+	-
**Laboratory findings**					
WBC	3,570	3,710	6,720	4,730	5,820
Hemoglobin	10.3	12.9	16.1	12.9	10.8
Platelet	157,000	42,000	190,000	316,000	175,000
AST	23	57	32	34	43
ALT	22	55	13	38	18
Creatinine	0.83	0.77	0.55	0.31	0.38
**Used drug**					
Lopinavir/ritonavir	+	+	+	+	+
Hydroxychloroquine	+	+	+	+	+
**Outcome**	Cured	Cured	Cured	Cured	Cured
**Ct value**	22.93	30.1	37.85	38.81	29.31
